# Effects of Initial Morphology on Growth Kinetics of Cu_6_Sn_5_ at SAC305/Cu Interface during Isothermal Aging

**DOI:** 10.3390/ma15144751

**Published:** 2022-07-07

**Authors:** Jia-Yi Lee, Chih-Ming Chen

**Affiliations:** 1 Department of Chemical Engineering, National Chung Hsing University, Taichung 402, Taiwan; junior30304@gmail.com; 2 Innovation and Development Center of Sustainable Agriculture (IDCSA), National Chung Hsing University, Taichung 402, Taiwan

**Keywords:** morphology, grain size, intermetallic compounds, thickness

## Abstract

Solder/Cu joints are important components responsible for interconnection in microelectronics. Construction of the solder/Cu joints through liquid/solid (L/S) reactions accompanies the formation of the Cu–Sn intermetallic compounds (IMCs) at the joint interface. The Cu_6_Sn_5_ IMC exhibits remarkable distinctions in thickness and morphology upon increasing the L/S reaction time. Effects of the initial characteristics of thickness and morphology on the growth kinetics of Cu_6_Sn_5_ during subsequent isothermal aging were investigated. SAC305 solder was reflowed on a Cu electroplated layer at 265 °C for 1 to 60 min to produce the Cu_6_Sn_5_ IMC with different thickness and morphology at the SAC305/Cu interface. The as-fabricated SAC305/Cu joint samples were aged at 200 °C for 72 to 360 h to investigate the growth kinetics of Cu_6_Sn_5_. The results show that the initial characteristics of thickness and morphology significantly influenced the growth kinetics of Cu_6_Sn_5_ during the subsequent solid/solid (S/S) reaction. A prolonged L/S reaction time of 60 min (L/S-60) produced a scallop-type Cu_6_Sn_5_ IMC with a larger grain size and a thicker thickness, which reduced the quantity of fast diffusion path (grain boundary) and the magnitude of concentration gradient, thus slowing down the growth rate of Cu_6_Sn_5_. According to the growth kinetics analysis, the growth rate constant of Cu_6_Sn_5_ could be remarkably reduced to 0.151 µm/h^0.5^ for the L/S-60 sample, representing a significant reduction of 70 % compared to that of the L/S-1 sample (0.508 µm/h^0.5^ for L/S reaction time of 1 min).

## 1. Introduction

Soldering is a joining technology widely used to construct the electrical connection and mechanical support in between heterogeneous components in microelectronic products. This joining technology is accomplished by means of the liquid/solid (L/S) reaction, commonly called the reflow reaction, at a proper temperature, during which a low-melting-point alloy melts and reacts with the metallization of high melting points on two adjoining components [1,2,3,4,5,6]. Sn-based alloys such as SnAg and SnAgCu have a moderate melting point around 217–220 °C, making them suitable as joining materials. Due to superior electrical and thermal properties, Cu is commonly used as the material for metallization in microelectronic products. Therefore, the liquid/solid reaction study of solder joints constructed using Sn-based alloy and Cu is an attractive and practically important issue [1,2,3,4,5,6,7,8,9,10,11].

Atomic interdiffusion occurs when Sn-based alloy is brought into contact with Cu, giving rise to the formation of intermetallic compounds (IMCs) such as Cu_6_Sn_5_ and Cu_3_Sn at the contact interface [12,13,14,15,16,17]. The cooling conditions after the liquid/solid reaction had a significant effect on the thickness and morphology of IMCs [18,19,20]. Water quenching produced a relatively planar Cu_6_Sn_5_ layer, whereas a scalloped Cu_6_Sn_5_ morphology was formed in the furnace cooling condition [18]. The differences in the thickness and interfacial morphology of initial Cu_6_Sn_5_ had a profound influence on the growth and evolution of IMCs in subsequent solid/solid (S/S) thermal aging [18]. Planarization took place in the scalloped Cu_6_Sn_5_ layer due to faster atomic diffusion in the scallop valleys in the furnace-cooled sample, while the Cu_6_Sn_5_ growth was dominated by bulk diffusion. The relatively planar Cu_6_Sn_5_ layer exhibited a mixed growth behavior dominated by grain boundary and bulk diffusion. 

The L/S reaction between Sn-based alloy and Cu is usually repeated several times in the assembly of microelectronic products that requires multiple levels of interconnection. The L/S reaction time also had a more significant effect on the thickness and morphology of IMCs compared to the cooling condition [18,21,22]. Moreover, an extension of the L/S reaction time could even magnify the differences in the thickness and morphology of IMCs as observed in the transient liquid phase bonding (TLP) process [23], giving rise to extremely different initial IMC characteristics prior to solid-state aging.

In this study, the L/S reactions of the SnAgCu/Cu joints were performed for various lengths of time to alter the thickness and morphology of IMCs. The SnAgCu/Cu joints with different initial IMC characteristics were thermally aged to perform the S/S reaction, and the focus was placed on the influence of initial IMC characteristics on the growth and morphological evolution of IMCs during the S/S thermal aging history. The Cu_6_Sn_5_ IMC grew thicker and the grain size augmented upon increasing the L/S reaction time, and this morphological evolution reduced the quantity of fast diffusion paths (grain boundaries) and the magnitude of the concentration gradient. As a result, the growth rate of Cu_6_Sn_5_ was reduced. The growth kinetics analysis revealed that the growth rate constant of Cu_6_Sn_5_ during the S/S reaction was remarkably reduced by 70% when the L/S reaction time was increased from 1 min to 60 min.

## 2. Experimental Procedures

The substrate used to prepare the solder joints was a Cu electroplated layer deposited on a 1 cm × 3 cm Cu foil (99.99 wt.%, UMAT Co., Hsinchu, Taiwan). The electrodeposition of Cu was performed in a Haring cell containing an electrolyte of high-purity CuSO_4_⋅5H_2_O, H_2_SO_4_, and specific additives. The specific additives were suppressor (polyethylene glycol, PEG, 2000 g/mole, 50 ppm), accelerator (bis(3-sulfopropyl) disulfide, SPS, 1 ppm), and chloride ions (NaCl, 60 ppm). They were added in the plating solutions to reduce the impurity concentrations in the Cu electroplated layer and to regulate the deposition behaviors of reduced Cu atoms. Furthermore, the Cu foil was placed on the cathode side to grow the Cu electroplated layer and a 0.04 wt.% phosphorus-containing Cu plate (UMAT Co., Hsinchu, Taiwan) was used as the anode. During the electroplating process for 1 h, the current density was kept as constant at 3.44 ASD, and the temperature of the Haring cell was controlled at 28 °C. After electroplating, the Cu substrate was rinsed using deionized water and dried by an air gun. To define the reaction area with the solder alloy, a heat-resistant tape (solder mask) with an aperture of 1.8 mm diameter was attached on the Cu electroplated layer.

**Figure 1:**
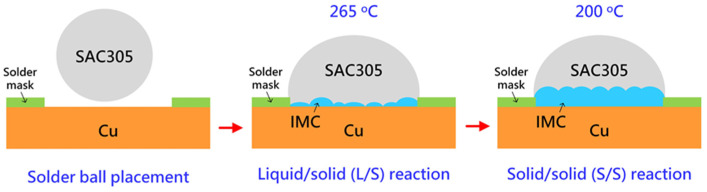
Schematic drawing of the preparation and configuration of the SAC305/Cu joints for liquid/solid and solid/solid reactions.

Commercially available SAC305 (Sn—3 wt.% Ag—0.5 wt.% Cu, Senju Metal Industry Co., Ltd., Tokyo, Janan) was used as the solder alloy. Proper amounts of alloy ingots of 22 mg were weighed and heated on a hot plate at 250 °C, during which the alloy ingots melted and transformed into a tiny ball. The tiny solder ball was coated with flux and placed on the exposed surface of Cu defined by the aperture of the heat-resistant tape. The liquid/solid (L/S) reaction between SAC305 and Cu was performed by placing the samples on a hot plate at 265 °C for 1, 10, 30, and 60 min. The as-joined SAC305/Cu samples for various L/S reaction times were denominated as L/S-1, L/S-10, L/S-30, and L/S60, where the number after L/S represents the reaction time. After the L/S reaction, the SAC305/Cu samples were placed in an oven at 200 °C to perform the solid/solid (S/S) reaction for 72, 168, and 360 h. The preparation and reaction of the SAC305/Cu samples are schematically shown in Figure 1. 

After the S/S reactions, the SAC305/Cu samples were removed from the oven and cooled down to room temperature naturally. The aged SAC305/Cu samples were mounted in epoxy resin and were ground in a direction perpendicular to the SAC305/Cu interface using SiC sandpaper. The exposed SAC305/Cu interfacial zone was polished using fine Al_2_O_3_ suspensions to offer scratch-free microstructures for examinations using optical microscopy (OM, BX51, Olympus, Tokyo, Japan). Energy-dispersive X-ray spectroscopy (EDX) attached to scanning electron microscopy (SEM, UltraPlus, Zeiss, Oberkochen, Germany) was used to analyze the elemental compositions of the IMCs formed at the SAC305/Cu interface. The average thickness of the IMCs was determined by dividing the area of the IMC layers by the linear length of the SAC305/Cu interface using an image processing software.

## 3. Results and Discussion

**Figure 2:**
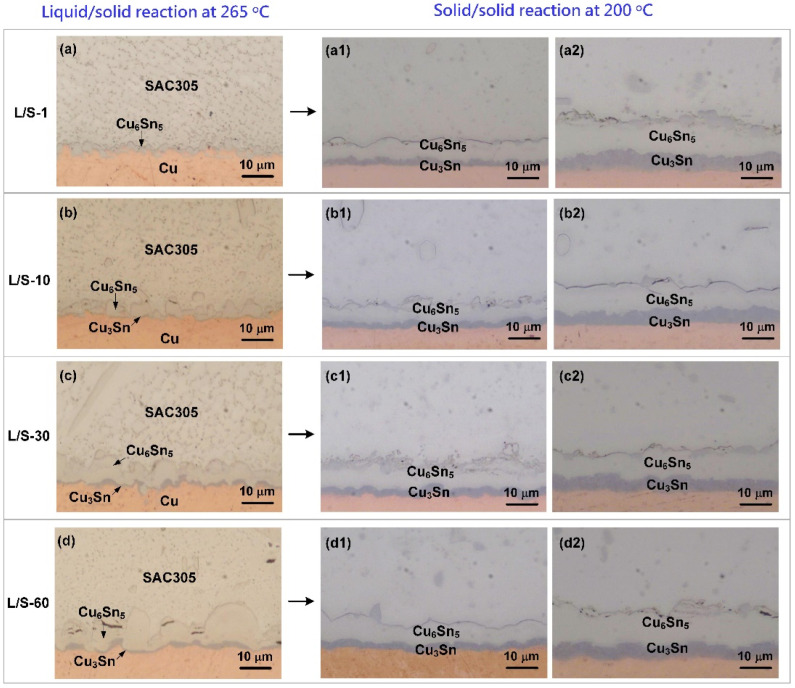
Cross-sectional OM micrographs of the cross-sections of the SAC305/Cu joints after the liquid/solid and solid/solid reactions. (**a**) 1 min; (**b**) 10 min; (**c**) 30 min; (**d**) 60 min; (**a1**–**d1**) 72 h; (**a2**–**d2**) 360 h.

There were four SAC305/Cu joint samples obtained from the L/S reaction, and they were denominated as L/S-1, L/S-10, L/S-30, and L/S-60, for various reaction times from 1 min to 60 min. Figure 2a–d show the OM micrographs of the cross-sections of the four SAC305/Cu joints after the L/S reaction. Only one IMC was formed at the SAC305/Cu interface after L/S reaction for 1 min as seen in Figure 2a, and it was identified as the Cu_6_Sn_5_ phase according to the EDX results (57.8 at.% Cu, 42.2 at.% Sn). A new IMC was formed at the Cu_6_Sn_5_/Cu interface after longer L/S reaction times (10, 30, and 60 min) as seen in Figure 2b–d, and it was confirmed as the Cu_3_Sn phase according to the EDX results (74.8 at.% Cu, 25.2 at.% Sn). The phase formation of Cu_6_Sn_5_ and Cu_3_Sn at the SAC305/Cu interface subjected to L/S reaction was consistent with previous studies [5,24]. For the Cu_6_Sn_5_ phase in the early stage of L/S reaction (L/S-1 sample) in Figure 2a, it grew in the form of small granules at the interface, and its average thickness was 2.65 µm. The Cu_6_Sn_5_ phase in the later stage of L/S reaction (L/S-60 sample) in Figure 2d grew thicker (6.9 µm in the average thickness) and exhibited a scallop-shaped morphology. Due to the scallop-shaped morphology, the thickness of the Cu_6_Sn_5_ phase was uneven; some Cu_6_Sn_5_ scallops grew to be as thick as 15 µm in the direction perpendicular to the interface, while some were 10 µm in the thickness. The Cu_6_Sn_5_ phase formed at the valleys in between two neighboring scallops was less than 5 µm thick. In the other two samples (L/S-10 and L/S-30), as seen in Figure 2b,c, the Cu_6_Sn_5_ phase also grew into a scallop-shaped structure, while the scallop size was smaller.

**Figure 3:**
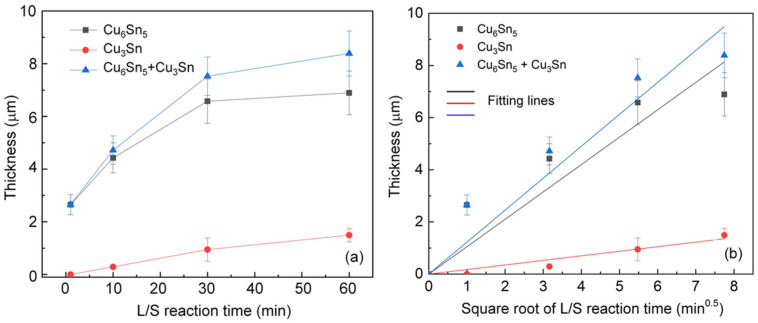
Thicknesses of IMCs as a function of (**a**) the liquid/solid reaction time and (**b**) the square root of the reaction time.

The average thickness of IMCs formed in the L/S joint samples was measured and plotted as a function of the reaction time as shown in Figure 3a. Overall, the thicknesses of the IMCs were found to increase with increasing reaction time. In general, the thickness of IMC can be formulated as a function of the square root of reaction time by assuming a diffusion-controlled growth mechanism [20].

Δ*x* = *x* − *x*_0_ = *k t*^0.5^, (1)

where *x*, *x*_0_, *k*, and *t* are the IMC thickness after the reaction, the initial IMC thickness before the reaction, the growth rate constant, and the reaction time, respectively. By plotting the relationship between Δ*x* and *t*^0.5^ as shown in Figure 3b, the growth rate constant (*k*) can be determined from the slope of the fitting line according to Equation (1). The values of *k* for the IMCs formed in the L/S reactions are listed in Table 1. It was found that the growth rate constant of Cu_6_Sn_5_ was 1.05 µm/min^0.5^, which was approximately six times larger than that of Cu_3_Sn (0.175 µm/min^0.5^). The growth of Cu_3_Sn was generally governed by the phase transformation described below.

Cu_6_Sn_5_ + 9 Cu → 5 Cu_3_Sn. (2)

Because the phase transformation involved the solid-state reaction between Cu_6_Sn_5_ and Cu, the Cu_3_Sn phase was expected to have a smaller growth rate constant compared with the Cu_6_Sn_5_ phase, which was formed as a result of the liquid/solid reaction.

**Figure 4:**
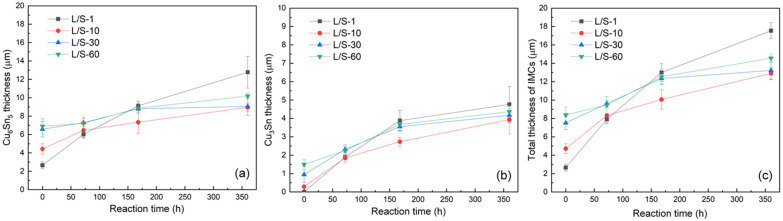
Thicknesses of the IMCs as a function of the solid/solid reaction time for various liquid/solid joints: (**a**) Cu_6_Sn_5_, (**b**) Cu_3_Sn, and (**c**) Cu_6_Sn_5_ + Cu_3_Sn.

**Figure 5:**
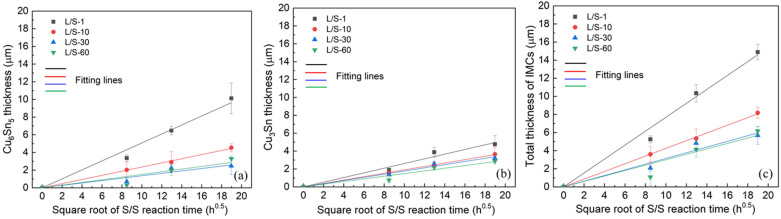
Thicknesses of the IMCs as a function of the square root of reaction time for various liquid/solid joints: (**a**) Cu_6_Sn_5_, (**b**) Cu_3_Sn, and (**c**) Cu_6_Sn_5_ + Cu_3_Sn.

After the L/S reactions, the four SAC305/Cu joint samples were thermally aged at 200 °C to perform the S/S reaction for various lengths of time. As shown in Figure 2(a1–d2), Cu_6_Sn_5_ and Cu_3_Sn were observed at the interfaces of the four samples, and both displayed a uniform layer-type structure. The morphological change from the scallop type (L/S reaction) into layer type (S/S reaction) was accomplished by faster atomic diffusion at the scallop valleys, which accelerated the IMC growth there to catch up with the growth front at the scallop peaks, resulting in the planarization of the Cu_6_Sn_5_ scallops [18]. Figure 4 depicts the thicknesses of the IMCs in the four samples after S/S reaction for various lengths of time. Both Cu_6_Sn_5_ and Cu_3_Sn grew thicker with increasing reaction time as shown in Figure 4a,b, respectively, was as did the total IMCs (Cu_6_Sn_5_ + Cu_3_Sn), as shown in Figure 4c. The thickness data of the IMCs were also plotted as a function of the square root of reaction time, as shown in Figure 5. The fitting results based on Equation (1) indicated that the growth of all IMCs obeyed the parabolic law, and this growth was diffusion-controlled. Table 2 lists the magnitudes of *k* for the growth of IMCs in the four L/S samples during the S/S reactions. The magnitude of *k* for Cu_6_Sn_5_ formed in the L/S-1 sample was 0.508 µm/h^0.5^, but it decreased to 0.235 µm/h^0.5^, 0.136 µm/h^0.5^, and 0.151 µm/h^0.5^ for the L/S-10, L/S-30, and L/S-60 samples, respectively. The decreasing trend of *k* was also observed for the Cu_3_Sn phase, as well as the total IMCs (Cu_6_Sn_5_ + Cu_3_Sn). This phenomenon indicated that the initial characteristics of IMCs (mainly Cu_6_Sn_5_) played an important role in the growth behavior of IMCs in the thermal aging process. When the initial morphology of the Cu_6_Sn_5_ phase was thin and of the layer type (L/S-1), it grew at a faster rate with a higher *k* of 0.508 µm/h^0.5^. However, the growth of the Cu_6_Sn_5_ phase with an initial scallop-shaped morphology (L/S-60) was sluggish due to a lower *k* of 0.151 µm/h^0.5^. 

**Figure 6:**
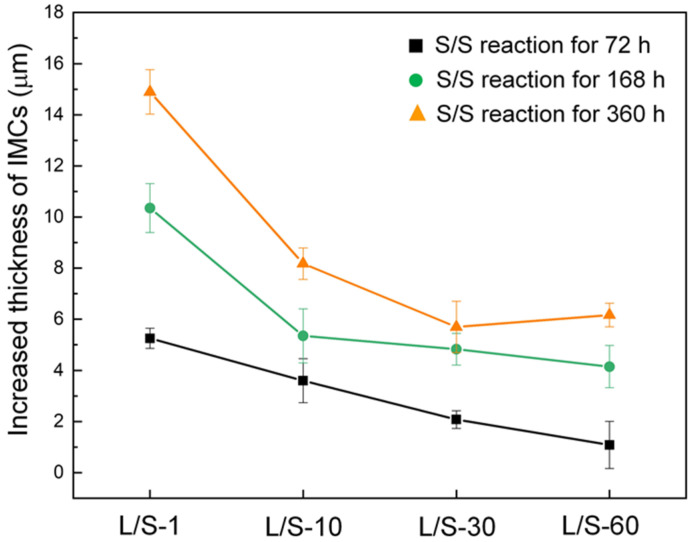
Increased thickness of the IMCs for various L/S reaction samples during the S/S reaction.

Figure 6 depicts the increment in total thickness of the IMCs for the four L/S samples during the S/S reaction. For the L/S-1 sample, the thickness of IMCs increased by 5 µm, 10 µm, and 15 µm after the S/S reaction for 72 h, 168 h, and 360 h, respectively. The increased thicknesses of IMCs were only 1 µm, 4 µm, and 6 µm for the L/S-60 sample after the same S/S reaction times, which were reduced by 80%, 60%, and 60%, respectively, compared to those in the L/S-1 sample. As mentioned above, the Cu_6_Sn_5_ phase predominated the growth of the IMCs at the SAC305/Cu interface due to its faster growth rate. For simplicity, only the Cu_6_Sn_5_ phase was taken into consideration in the discussion of the growth of IMCs. In addition, Sn was considered as the faster diffusion species that controlled the growth of Cu_6_Sn_5_ at 200 °C [12]. Therefore, the growth of the IMCs (Cu_6_Sn_5_) was governed by the atomic diffusion flux, as described by Equation (3).

**Figure 7:**
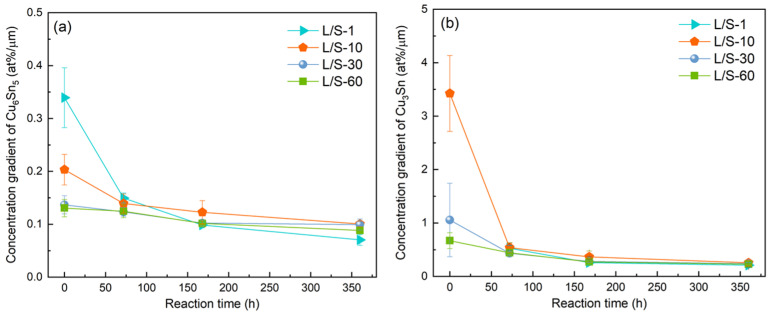
Magnitudes of |Δ*C_Sn_*/Δ*x*| (absolute value) of (**a**) Cu_6_Sn_5_ and (**b**) Cu_3_Sn as a function of the S/S reaction time.

*J_Sn_* = −*D_Sn_* × (*dC_Sn_*/*dx*), (3)

where *J_Sn_*, *D_Sn_*, *C_Sn_*, and *x* are the diffusion flux of Sn, the diffusivity of Sn, the concentration of Sn, and the distance along the diffusion direction, respectively. According to Equation (3), the growth rate of Cu_6_Sn_5_ is influenced by two parameters; one is the diffusivity of Sn (*D_Sn_*), and the other is the concentration gradient (*dC_Sn_*/*dx*). The latter parameter, *dC_Sn_*/*dx*, can be calculated for quantitative comparison. Assuming a linear concentration gradient within the Cu_6_Sn_5_ phase, the magnitude of *dC_Sn_*/*dx* is equal to Δ*C_Sn_*/Δ*x*, where Δ*C_Sn_* is the concentration difference of Sn at the two boundaries of the Cu_6_Sn_5_ layer, and Δ*x* is the thickness of Cu_6_Sn_5_. Δ*C_Sn_* can be calculated from the homogeneity range information of Cu_6_Sn_5_ in the Sn–Cu phase diagram with the assumption of a local equilibrium [25]. At 200 °C, the homogeneity range of Cu_6_Sn_5_ ranged from 43.4 at.% Sn at the Cu_3_Sn/Cu_6_Sn_5_ interface to 44.3 at.% Sn at the Cu_6_Sn_5_/Sn interface; thus, Δ*C_Sn_* was calculated to be −0.9 at.%, which is a constant independent of the IMC thickness. In the L/S-1 sample, the thickness of initial Cu_6_Sn_5_ was measured to be 2.65 µm, as shown in Figure 4a. Then, Δ*C_Sn_*/Δ*x* was determined to be −0.34 at.%/µm for the initial Cu_6_Sn_5_ phase prior to the S/S reaction. Following a similar calculation process, the magnitudes of |Δ*C_Sn_*/Δ*x*| (absolute value) of the Cu_6_Sn_5_ phase at the different stages of S/S reaction for all L/S reaction samples were determined as listed in Table 3. Their relationship with the reaction time is plotted in Figure 7. 

As shown in Figure 7, all L/S samples displayed a decreasing trend of |Δ*C_Sn_*/Δ*x*| with increasing reaction time due to an increasing Δ*x*. As expected, the L/S-1 sample had a larger value of |Δ*C_Sn_*/Δ*x*| at the initial stage of the S/S reaction due to a smaller Δ*x*. A larger value of |Δ*C_Sn_*/Δ*x*| gave rise to a larger *J_Sn_*, which accelerated the growth of Cu_6_Sn_5_ in the L/S-1 sample (Figure 4). With increasing S/S reaction time, the thickness of Cu_6_Sn_5_ increased; hence, the magnitude of |Δ*C_Sn_*/Δ*x*| decreased. When the reaction time reached 72 h, the |Δ*C_Sn_*/Δ*x*| of the L/S-1 sample decreased to a level very close to those of the other three samples (L/S-10, L/S-30, and L/S-60) due to the high similarity in the IMC thickness. After the S/S reaction for 168 h and 360 h, the values of |Δ*C_Sn_*/Δ*x*| for all four L/S samples were very similar to each other, as shown in Figure 7. It was expected that the growth rates of the IMCs in the four L/S samples would differ very slightly after S/S reaction for 168 h due to the high similarity of |Δ*C_Sn_*/Δ*x*| and *J_Sn_*. However, in the L/S-1 sample, the IMCs continued to grow, and the thickness exceeded that of the other samples after S/S reaction for 168 h, as shown in Figure 4. The discrepancy can be explained from the perspective of grain microstructures of IMCs. 

**Figure 8:**
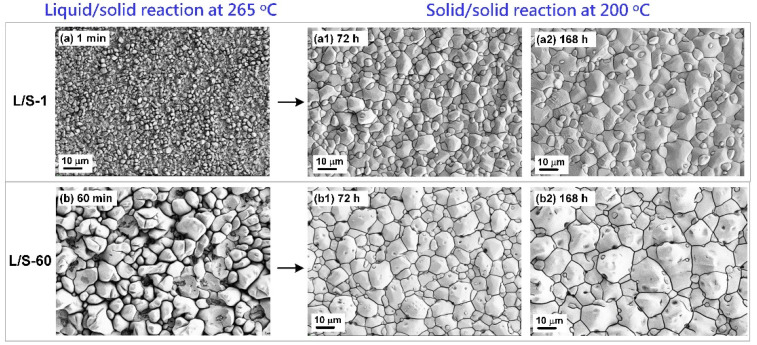
Top-view SEM micrographs of the Cu_6_Sn_5_ grains in the L/S-1 (**a**,**a1**,**a2**) and L/S-60 (**b**,**b1**,**b2**) samples subjected to S/S reaction for various lengths of time.

**Figure 9:**
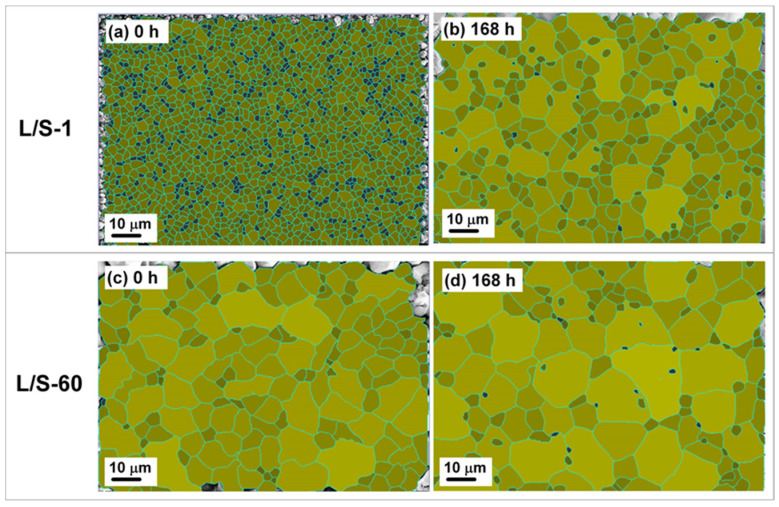
Size measurement of the Cu_6_Sn_5_ grains in the L/S-1 (**a**,**b**) and L/S-60 (**c**,**d**) samples subjected to S/S reaction for various lengths of time.

Figure 8 shows the top-view SEM micrographs of the SAC305/Cu interfaces in the L/S samples subjected to the S/S reaction for various lengths of time. The solder portion was entirely removed using an etching solution (10% HNO_3_) to expose the Cu_6_Sn_5_ grains for three-dimensional observation. Before the S/S reaction, the Cu_6_Sn_5_ grains in the L/S samples displayed a granular (scallop) microstructure with a smaller grain size in the L/S-1 sample but a much larger grain size in the L/S-60 sample. After the S/S reaction, planarization of the Cu_6_Sn_5_ grains occurred by means of faster atomic diffusion in the scallop valleys. Grain ripening and growth also occurred, which was evidenced by the presence of some tiny grains surrounding giant grains in both samples. The average grain sizes in the L/S-1 and L/S-60 samples after S/S reaction for 168 h were measured to be 27.2 µm^2^ and 54.7 µm^2^, respectively, using an image processing software as shown in Figure 9. The grain size in the L/S-1 sample was smaller than that in the L/S-60 sample, meaning a higher grain boundary quantity in the Cu_6_Sn_5_ layer of the L/S-1 sample, which might have influenced the atomic diffusion behavior. 

The apparent diffusion coefficient (*D_app_*) of Sn in the Cu_6_Sn_5_ layer can be formulated by Equation (4) [26].

*D_app_* = *D_l_* + *D_b_* (*δ*/*d*), (4)

where *D_l_* and *D_b_* are the volume and grain boundary diffusion coefficients, respectively, and *δ* and *d* are the grain boundary thickness and grain size of Cu_6_Sn_5_, respectively. The ratio of *δ*/*d* is an indicator that can reflect the contribution of grain boundary diffusion to the total atomic diffusion. Because the Cu_6_Sn_5_ phase in the L/S-1 sample had a smaller grain size (*d*) compared to that in the L/S-60 sample, the grain boundary diffusion should have made a greater contribution in the L/S-1 sample than in the L/S-60 sample. As a result, the magnitude of *D_app_* was enhanced in the L/S-1 sample, giving rise to an enhanced atomic flux (*J_Sn_*), as well as a faster growth rate of the Cu_6_Sn_5_ phase. In contrast, the grain size (*d*) in the L/S-60 sample was larger, which resulted in a smaller size ratio (*δ*/*d*) and weakened the contribution of grain boundary diffusion.

**Figure 10:**
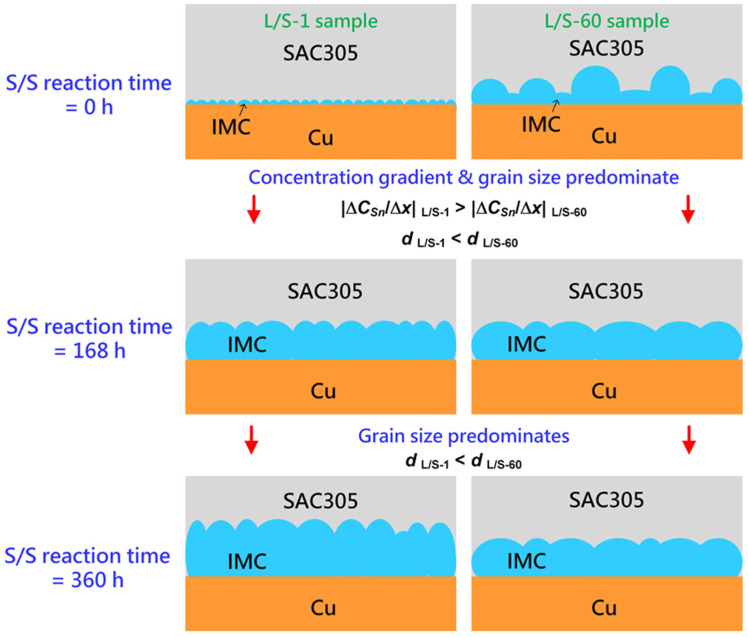
Schematic drawing of the IMC growth behavior in the L/S-1 and L/S-60 samples subjected to the S/S reactions.

Figure 10 shows a schematic drawing of the IMC growth in the two L/S samples (L/S-1 and L/S-60) which had a very different initial thickness and morphology of IMC (mainly Cu_6_Sn_5_) before performing the S/S reaction. At the initial stage of the S/S reaction (t = 0 h), the Cu_6_Sn_5_ phase was thin (2.65 µm) in the L/S-1 sample but was thicker (6.9 µm) in the L/S-60 sample. In addition, the grain size of Cu_6_Sn_5_ was extremely different in the two samples. The Cu_6_Sn_5_ grains were much smaller in the L/S-1 sample (4.5 µm^2^) than in the L/S-60 sample (46.3 µm^2^) as shown in Figure 9. When the two samples were subjected to thermal aging at 200 °C (S/S reaction), the Cu_6_Sn_5_ phase continued to grow due to atomic interdiffusion occurring at the SAC305/Cu interface. During the early stage of S/S reaction (t = 0–168 h), the atomic interdiffusion was enhanced in the L/S-1 sample due to a synergy resulting from a higher concentration gradient (|Δ*C_Sn_*/Δ*x*|) and a higher apparent diffusion coefficient (*D_app_*). As a result, the growth rate of Cu_6_Sn_5_ was enhanced in the L/S-1 sample. Even though the initial Cu_6_Sn_5_ phase was thin in the L/S-1 sample, it could catch up that of the L/S-60 sample, which had a thicker initial Cu_6_Sn_5_ phase. By contrast, the growth rate of Cu_6_Sn_5_ was sluggish in the L/S-60 sample due to a lower concentration gradient (|Δ*C_Sn_*/Δ*x*|) and a lower apparent diffusion coefficient (*D_app_*). In the later stage of S/S reaction (t = 168–360 h), the influence of concentration gradient was similar in the two samples. However, the Cu_6_Sn_5_ phase still had a smaller grain size in the L/S-1 sample than in the L/S-60 sample, indicating that the apparent diffusion coefficient should have been higher in the L/S-1 sample. Therefore, the Cu_6_Sn_5_ phase in the L/S-1 sample still grew at a faster rate than that in the L/S-60 sample as seen in Figure 4a. In summary, the initial characteristics of thickness and morphology significantly influenced the growth kinetics of Cu_6_Sn_5_ during the S/S reaction. According to the growth kinetics analysis (Table 2), the growth rate constant of Cu_6_Sn_5_ was remarkably reduced to 0.151 µm/h^0.5^ for the L/S-60 sample, which was a significant reduction of 70% compared to that of the L/S-1 sample (0.508 µm/h^0.5^).

## 4. Conclusions

SAC305 solder was joined with Cu by means of the L/S reaction at 265 °C. The Cu_6_Sn_5_ IMC was formed at the SAC305/Cu interface and exhibited distinct characteristics in the thickness and morphology during the course of the L/S reactions. A layer-type Cu_6_Sn_5_ IMC with a thickness of 2.65 µm and a grain area of 4.5 µm^2^ was produced after a shorter L/S reaction time of 1 min, while the Cu_6_Sn_5_ IMC evolved into scallop-type IMC with a thickness of 6.9 µm and a grain area of 46.3 µm^2^ after a longer L/S reaction time of 60 min. During the subsequent S/S reactions, the Cu_6_Sn_5_ IMC with a coarse initial characteristic (thicker thickness and larger grain size) grew at a slower rate compared to that with a fine initial characteristic (thinner thickness and smaller grain size). In the early stage of the S/S reaction, the slower growth rate was attributed to the thicker thickness of IMC, giving rise to a lower driving force of concentration gradient and, therefore, a lower atomic flux. The larger grain morphology offered a lower density of grain boundary for atomic diffusion, also contributing to the slower growth rate of Cu_6_Sn_5_. However, the influence of concentration gradient became insignificant in the later stage due to the high similarity in the IMC thickness. Nonetheless, the grain morphology became the main influencing factor, and the Cu_6_Sn_5_ IMC with a coarser grain size still grew at a slower rate due to retardant grain boundary diffusion.

## Figures and Tables

**Table 1:** Growth rate constants (*k*) of Cu_6_Sn_5_, Cu_3_Sn, and Cu_6_Sn_5_ + Cu_3_Sn for the liquid/solid reactions at 265 °C.

	Cu_6_Sn_5_	Cu_3_Sn	Cu_6_Sn_5_ + Cu_3_Sn
*k* (µm/min^0.5^)	1.05 ± 0.122	0.175 ± 0.017	1.225 ± 0.107

**Table 2:** Growth rate constants (*k*) of Cu_6_Sn_5_, Cu_3_Sn, and Cu_6_Sn_5_ + Cu_3_Sn for thermal aging (solid/solid) reactions at 200 °C (unit: µm/h^0.5^).

Samples	Cu_6_Sn_5_	Cu_3_Sn	Cu_6_Sn_5_ + Cu_3_Sn
L/S-1	0.508 ± 0.025	0.261 ± 0.015	0.769 ± 0.032
L/S-10	0.235 ± 0.004	0.19 ± 0.002	0.425 ± 0.005
L/S-30	0.136 ± 0.015	0.178 ± 0.009	0.314 ± 0.023
L/S-60	0.151 ± 0.025	0.149 ± 0.013	0.3 ± 0.037

**Table 3:** Magnitude of concentration gradient of Cu_6_Sn_5_ and Cu_3_Sn for various L/S samples subjected to thermal aging at 200 °C for various lengths of S/S reaction time. (unit: at.%/µm).

	Cu_6_Sn_5_				Cu_3_Sn			
	0 h	72 h	168 h	360 h	0 h	72 h	168 h	360 h
L/S-1	0.34	0.15	0.097	0.07	-	0.528	0.258	0.21
L/S-10	0.203	0.139	0.123	0.1	3.425	0.538	0.366	0.254
L/S-30	0.137	0.124	0.102	0.099	1.056	0.432	0.282	0.24
L/S-60	0.131	0.124	0.102	0.088	0.669	0.444	0.272	0.229

## Data Availability

All of the data supporting the reported results can be found in this manuscript.

## References

[1] Laurila T., Vuorinen V., Paulasto-Kröckel M. (2010). Impurity and alloying effects on interfacial reaction layers in Pb-free soldering. Mater. Sci. Eng. R Rep..

[2] Chen C.J., Chen C.M., Horng R.H., Wuu D.S., Hong J.S. (2010). Thermal management and interfacial properties in high-power GaN-based light-emitting diodes employing diamond-added Sn-3 wt.%Ag-0.5 wt.%Cu solder as die attach materials. J. Electron. Mater..

[3] Liu T.C., Liu C.M., Huang Y.S., Chen C., Tu K.N. (2013). Eliminate Kirkendall voids in solder reactions on nanotwinned copper. Scr. Mater..

[4] Ho C.E., Hsu L.H., Yang C.H., Yeh T.C., Lee P.T. (2015). Effect of Pd(P) thickness on the soldering reaction between Sn–3Ag–0.5Cu alloy and ultrathin-Ni(P)-type Au/Pd(P)/Ni(P)/Cu metallization pad. Thin Solid Film..

[5] Cheng H.K., Huang C.W., Lee H., Wang Y.L., Liu T.F., Chen C.M. (2015). Interfacial reactions between Cu and SnAgCu solder doped with minor Ni. J. Alloy. Compd..

[6] Gusak A.M., Tu K.N., Chen C. (2020). Extremely rapid grain growth in scallop-type Cu6Sn5 during solid–liquid interdiffusion reactions in micro-bump solder joints. Scr. Mater..

[7] Hsu H.L., Lee H., Wang C.W., Liang C., Chen C.M. (2019). Impurity evaporation and void formation in Sn/Cu solder joints. Mater. Chem. Phys..

[8] Wang H., Zhang K., Zhang M. (2019). Fabrication and properties of Ni-modified graphene nanosheets reinforced Sn-Ag-Cu composite solder. J. Alloy. Compd..

[9] Tu K.N., Liu Y. (2019). Recent advances on kinetic analysis of solder joint reactions in 3D IC packaging technology. Mater. Sci. Eng. R Rep..

[10] Shen Y.A., Zhou S., Li J., Yang C.H., Huang S., Lin S.K., Nishikawa H. (2019). Sn-3.0Ag-0.5Cu/Sn-58Bi composite solder joint assembled using a low-temperature reflow process for PoP technology. Mater. Des..

[11] He S., Gao R., Li J., Shen Y.A., Nishikawa H. (2020). In-situ observation of fluxless soldering of Sn-3.0Ag-0.5Cu/Cu under a formic acid atmosphere. Mater. Chem. Phys..

[12] Yu T.Y., Lee H., Hsu H.L., Dow W.P., Cheng H.K., Liu K.C., Chen C.M. (2016). Effects of Cu electroplating formulas on the interfacial microstructures of Sn/Cu joints. J. Electrochem. Soc..

[13] Yang T.L., Aoki T., Matsumoto K., Toriyama K., Horibe A., Mori H., Orii Y., Wu J.Y., Kao C.R. (2016). Full intermetallic joints for chip stacking by using thermal gradient bonding. Acta Mater..

[14] Ho C.E., Yang S.P., Lee P.T., Lee C.Y., Chen C.C., Kuo T.T. (2021). IMC microstructure modification and mechanical reinforcement of Sn–Ag–Cu/Cu microelectronic joints through an advanced surface finish technique. J. Mater. Res. Technol..

[15] Ramli M.I.I., Salleh M.A.A.M., Abdullah M.M.A.B., Zaimi N.S.M., Sandu A.V., Vizureanu P., Rylski A., Amli S.F.M. (2022). Formation and Growth of Intermetallic Compounds in Lead-Free Solder Joints: A Review. Materials.

[16] Kao C.-W., Kung P.-Y., Chang C.-C., Huang W.-C., Chang F.-L., Kao C.R. (2022). Highly Robust Ti Adhesion Layer during Terminal Reaction in Micro-Bumps. Materials.

[17] Zaimi N.S.M., Salleh M.A.A.M., Abdullah M.M.A.-B., Nadzri N.I.M., Sandu A.V., Vizureanu P., Ramli M.I.I., Nogita K., Yasuda H., Sandu I.G. (2022). Effect of Kaolin Geopolymer Ceramics Addition on the Microstructure and Shear Strength of Sn-3.0Ag-0.5Cu Solder Joints during Multiple Reflow. Materials.

[18] Deng X., Piotrowski G., Williams J.J., Chawla N. (2003). Influence of Initial Morphology and Thickness of Cu6Sn5 and Cu3Sn Intermetallics on Growth and Evolution during Thermal Aging of Sn-Ag Solder/Cu Joints. J. Electron. Mater..

[19] Wang J.Y., Lin C.F., Chen C.M. (2011). Retarding the Cu5Zn8 phase fracture at the Sn-9 wt.% Zn/Cu interface. Scr. Mater..

[20] Hu X., Xu T., Jiang X., Li Y., Liu Y., Min Z. (2016). Effects of post-reflow cooling rate and thermal aging on growth behavior of interfacial intermetallic compound between SAC305 solder and Cu substrate. Appl. Phys. A.

[21] Deng X., Sidhu R.S., Johnson P., Chawla N. (2005). Influence of reflow and thermal aging on the shear strength and fracture behavior of Sn-3.5Ag solder/Cu joints. Metall. Mater. Trans. A.

[22] Gong S., Chen G., Qu S., Ren A., Duk V., Shi Q., Zhang G. (2021). Shear strength and fracture analysis of Sn-9Zn-2.5Bi-1.5In and Sn-3.0Ag-0.5Cu pastes with Cu-substrate joints under different reflow times. Microelectron. Reliab..

[23] Li J.F., Agyakwa P.A., Johnson C.M. (2011). Interfacial reaction in Cu/Sn/Cu system during the transient liquid phase soldering process. Acta Mater..

[24] Yang M., Ji H., Wang S., Ko Y.H., Lee C.W., Wu J., Li M. (2016). Effects of Ag content on the interfacial reactions between liquid Sn-Ag-Cu solders and Cu substrates during soldering. J. Alloy. Compd..

[25] Fürtauer S., Li D., Cupid D., Flandorfer H. (2013). The Cu–Sn phase diagram, Part I: New experimental results. Intermetallics.

[26] Porter D.A., Easterling K.E. (1992). Phase Transformations in Metals and Alloys.

